# Comparing the Associations of Dietary Patterns Identified through Principal Component Analysis and Cluster Analysis with Colorectal Cancer Risk: A Large Case–Control Study in China

**DOI:** 10.3390/nu16010147

**Published:** 2023-12-31

**Authors:** Ting Ma, Kexin Tu, Qingjian Ou, Yujing Fang, Caixia Zhang

**Affiliations:** 1 Department of Epidemiology, School of Public Health, Sun Yat-sen University, Guangzhou 510080, China; mating9@mail2.sysu.edu.cn (T.M.); tukx@mail2.sysu.edu.cn (K.T.); 2 State Key Laboratory of Oncology in South China, Guangdong Provincial Clinical Research Center for Cancer, Sun Yat-sen University Cancer Center, Guangzhou 510060, China

**Keywords:** dietary pattern, colorectal cancer, principal component analysis, cluster analysis

## Abstract

Examining the association between dietary patterns and colorectal cancer (CRC) risk can provide valuable insights beyond the assessment of individual foods or nutrients. However, there is a lack of in-depth analysis of dietary patterns and CRC risk in Chinese populations, and few studies have compared dietary patterns derived from different posteriori methods with the aim of predicting disease risk. The aim of this study was to derive dietary patterns using both principal component analysis (PCA) and cluster analysis (CA) and to assess their respective associations with CRC risk. A large-scale case-control study was conducted in Guangdong Province, China, including 2799 incident colorectal cancer cases and an equal number of frequency-matched controls. Dietary intake information was gathered through the use of a validated food frequency questionnaire. PCA and CA were used to derive dietary patterns. A multivariable logistic regression model was used to calculate the adjusted odds ratio (aOR) and 95% confidence interval (CI). Four major dietary patterns were identified by PCA. CA identified two dietary patterns, referred to as the “Balanced dietary pattern” and the “Refined grain dietary pattern”. Notably, there were significant inverse associations between the milk-egg-nut-soy dietary pattern (aOR, 0.51; 95% CI, 0.42, 0.62), the vegetable-fruit dietary pattern (aOR, 0.61; 95%CI, 0.51, 0.74), and the poultry-fish dietary pattern (aOR, 0.81; 95%CI, 0.68, 0.97) and CRC risk. However, the red meat-preserved food dietary pattern was associated with an increased risk of CRC (aOR, 2.99; 95%CI, 2.43, 3.67). When compared with the Refined grain dietary pattern, the Balanced dietary pattern showed a decreased risk of CRC (aOR, 0.59; 95%CI, 0.52, 0.66). The results from the comparison of the two methods indicate that both CA and PCA derived remarkably similar patterns. The combined use of PCA and CA identified consistent underlying patterns, showing comparable associations with CRC risk. These findings suggest that individuals who prefer dietary patterns characterized by a high intake of red meat, preserved food, and refined grains should be cautious about their increased CRC risk. Conversely, dietary patterns rich in fruits, vegetables, and high-quality protein sources are advisable for the prevention of CRC in the Chinese population.

## 1. Introduction

Colorectal cancer (CRC) is a global public health problem. According to the latest cancer statistics, CRC ranked as the second leading cause of cancer-related mortality worldwide and the third most common cancer diagnosed in 2020 [1]. There is substantial evidence suggesting that dietary and lifestyle changes may play an important role in the primary prevention of CRC [2]. While several studies have extensively explored the association of individual foods and nutrients with CRC risk, it is recognized that analyzing single food items or nutrients alone is insufficient to capture the intricate interactions among various nutrients [3,4]. Therefore, dietary pattern analysis serves as an alternative and complementary approach to identifying the connections between diet and disease risk.

Two commonly used empirical methods for food pattern analysis are principal component analysis (PCA) and cluster analysis (CA) [5,6,7,8]. PCA derives new dietary and food pattern variables by considering the correlation matrix of the original food variables and assigning a factor score to individuals for each of these derived factors. On the other hand, CA classifies individuals with similar dietary habits into exclusive subgroups based on the means of the food intake variables [6].

Several studies have explored the association between dietary patterns derived from PCA and CRC risk [9,10,11,12,13,14,15,16,17,18,19,20,21,22,23,24,25,26,27,28], but the results remain inconclusive. A Healthy dietary pattern, generally characterized by higher factor loadings of fruit, vegetables, whole grains, dairy products, poultry, and fish, is associated with a decreased risk of CRC in most cases [11,13,14,16,19,20,21,26], although some findings do not concur [9,10,18,22,23,24]. Conversely, an Unhealthy dietary pattern characterized by high consumption of red meat, preserved foods, and refined grains is frequently associated with an increased risk of CRC [11,12,15,16,17,18,25,26], but results from other studies may vary [27,28]. Three studies, conducted in Morocco [29], the United States [30], and France [31], used CA to identify dietary patterns and investigate their association with CRC risk. Each of these studies identified distinct clusters, and their associations with CRC varied as well. However, most of these studies investigating the association between CRC and dietary patterns were conducted in Western countries. To date, only two modest-sized case-control studies, one with 232 CRC cases and 232 controls and the other with 218 CRC cases and 218 controls, were carried out in North China in Shenyang and Qingdao [32,33], to explore the association between dietary patterns identified through PCA and the risk of CRC. Additionally, no study has employed CA to derive dietary patterns. Considering the variations in demographics and dietary preferences in different populations, it is essential to examine the potential correlations between dietary patterns and CRC risk in diverse Chinese populations.

Comparative analyses of dietary pattern methodologies are pivotal for understanding their strengths and limitations in nutrition research. In particular, employing both methods within a single dataset enhances our understanding of the similarities and differences between these approaches. However, there is a shortage of studies that directly compare the two methods, CA and PCA, when exploring the association between dietary patterns and CRC risk. Only one study from the United States did compare three dietary pattern methods—cluster analysis; factor analysis; and index analysis—in relation to CRC risk [34].

The main objective of this study encompassed two aspects: first, to derive the main dietary patterns among the Chinese population using PCA and CA, and second, to examine their associations with CRC risk. Second, to delve into disparities between dietary patterns derived through these two methods so as to provide more precise dietary recommendations for CRC prevention.

## 2. Materials and Methods

### 2.1. Study Subjects

This case-control study was initiated in July 2010 with the aim of examining the association between lifestyle factors and CRC risk in Guangdong, China. A detailed report of this study has been previously published [35,36]. In brief, potential cases between the ages of 30 and 75 were recruited from the surgical units of the Sun Yat-sen University Cancer Center in Guangzhou, China. Inclusion criteria included patients who were newly histologically diagnosed with primary CRC within the last 3 months and were either natives of Guangdong province or had lived in Guangdong for a minimum of 5 years. Patients diagnosed with other types of cancer or those who were unable to understand or speak Mandarin/Cantonese were excluded. From July 2010 to April 2021, a total of 2985 cases were identified, and 2833 eligible cases completed interviews, resulting in a response rate of 94.91%. An additional 34 patients were excluded due to extreme energy intake (<600 or >3500 kcal/d for women, <800 or >4200 kcal/d for men). Finally, 2799 CRC patients were included in the analysis.

Controls, devoid of any history of cancer, were frequency matched to the cases based on age (in 5-year intervals) and sex. One of the control groups was chosen from diverse departments, including Plastic and Reconstructive Surgery, Ophthalmology, Vascular Surgery, Otolaryngology, Orthopedics, and Microsurgery at the First Affiliated Hospital of Sun Yat-sen University, during the same time period as the cases. A total of 1738 hospital-derived controls were recruited, and 1499 successfully completed the interviews, yielding a participation rate of 86.25%. Another group of 1300 community-derived controls was obtained from residents residing in the same communities as the cases through referrals, invitations, and advertisements. The inclusion criteria for the control subjects were similar to those of the cases, except that they had no prior history of any cancers. Totally, the analysis included 2799 controls.

### 2.2. Data Collection

Professional interviewers conducted face-to-face interviews using a standardized questionnaire to obtain socio-demographic information, including age, sex, urban or rural residence, marital status, education, occupation, income, lifestyle factors such as smoking habits (both active and passive), alcohol consumption, physical activity, first-degree relatives with cancer, and anthropometric measurements including body height and weight. Female participants also provided information about their menarche age and menopausal status. Physical activity was assessed in terms of occupational activity and household/leisure-time activity. Occupational activity was classified into five levels based on labor intensity: non-working, sedentary, light occupation, moderate occupation, and heavy occupation activities. Metabolic equivalent (MET) values for housework and recreational activities were estimated using the Compendium of Physical Activities [37,38]. MET-hours per week over the past year were calculated as follows: the number of days per week multiplied by the number of hours per day multiplied by the MET for the specific type of activity, resulting in MET-hours per week. Body mass index (BMI) was calculated by dividing weight in kilograms by height in meters squared.

### 2.3. Dietary Assessment

A reliable and validated semi-quantitative food frequency questionnaire (FFQ) [39], comprising 81 items, was used to collect dietary intake information from all participants. The FFQ included 81 food and beverage items, including 12 types of cereals, 7 types of legumes, 18 types of vegetables, 11 types of fruits, 18 types of meat, 2 types of eggs, 8 types of dairy products, 3 types of beverages and soups, and 2 types of mushrooms and nuts. In total, there were 244 specific food items. Each participant was instructed to recall the usual frequency of consumption (per day, per week, per month, per year, or never) of various foods over the year preceding the diagnosis for cases or the interview for control subjects. A commonly used portion size (e.g., slice, glass, or unit, such as one apple or banana) was specified for each food item. In the case of vegetables and animal foods, liang (1 liang = 50 g), a commonly used unit in the study area familiar to the participants, was used to estimate their typical portion size. The daily intake of each food item (g/day) was calculated by multiplying the consumption frequency by the portion size. Food photographs displaying usual intake portions were provided to assist participants in estimating and recording their food consumption amounts. Total energy intake was computed using the China Food Composition Table 2002 [40].

### 2.4. Statistical Analysis

Data analysis was conducted with SPSS 25.0. (IBM Corporation, Armonk, NY, USA) and R version 4.3.0. *p* values < 0.05 were considered statistically significant. For enhanced interpretability of clusters and factors, the individual food items from the FFQ were initially aggregated into 13 food groups based on their nutrient profiles. Table 1 shows the food items included in each food group. The daily food intake data were log-transformed, and a residual method was used to adjust for energy [41]. The Wilcoxon rank-sum test was used for continuous variables, and the chi-square test was used for categorical variables to compare demographic and dietary intake variables between cases and controls.

Principal component factor analysis was conducted to derive dietary patterns using the daily consumption in grams of 13 food groups among control subjects only. Before applying PCA, the data’s suitability was assessed by conducting Bartlett’s test of sphericity and the Kaiser–Mayer–Olkin (KMO) test to ensure statistical correlation and sample size adequacy. Varimax rotation was used to simplify the structure and enhance interpretability. Factor retention for dietary patterns was determined by considering factors with a minimum eigenvalue of 1.0, the scree plot, and interpretability. The contribution of each food group to the pattern was measured through factor loadings, and patterns were named according to the major contributing food groups. Factor scores for each pattern represent the extent of alignment between the dietary intakes of the study subjects and the respective pattern. Higher factor scores indicate a stronger alignment.

For each dietary pattern, factor scores for the control group were divided into quintiles by sex. Based on the factor scores corresponding to these quintiles, all study participants were categorized into five groups, ranging from quintile1 (Q1) representing the lowest quintile to quintile5 (Q5) representing the highest quintile. A higher score on a factor of dietary pattern indicated a stronger adherence to that particular dietary pattern. A logistic regression model was used to estimate the risk of CRC using a dietary pattern quintile score, with the lowest quintile as the reference category. The *p* value for trend was calculated by treating Q1–Q5 as a continuous variable in the regression models. Potential confounders were chosen by comparing the characteristics of cases and controls for discrepancies. In the multivariable analysis, we adjusted for various factors, including age, sex, total energy intake, marital status, residence, educational level, occupation, income, BMI, smoking status, alcohol consumption, first-degree relative with cancer, occupational activity, and household and leisure-time activities.

Cluster analysis was performed to derive dietary patterns and classify participants based on the similarity of their diets using the k-means algorithm. The K-means cluster analysis, a non-hierarchical clustering technique, is carried out on the basis of Euclidean distances, thus positioning the cluster centers through least squares estimation. This analysis necessitates the pre-specification of the number of clusters. Several steps were taken to identify the most suitable number of clusters, with clustering being based on energy-adjusted and standardized food group variables. Initially, several runs were performed on the continuous food group variables, varying the number of clusters (ranging from two to eight). For each run, cluster proximities for each cluster center were examined, and the number of iterations per cluster was increased to ensure minimum error in cluster membership and ensure the model’s convergence. Clusters were also generated without outliers to help identify the optimal cluster solutions. Subsequently, we evaluated the solutions to determine which set of clusters best represented dietary patterns. The resulting clusters were numbered and provisionally labeled according to the food groups with significantly higher mean frequencies. To verify the reliability of the clustering results, we conducted a 50% random split of the sample and repeated the clustering process for each of the two subsets. Finally, a logistic regression model was used to estimate the risk of CRC with dietary patterns, using the larger cluster as a reference.

Differences between the two sets of patterns (components and clusters) were explored by comparing their food group composition and by plotting the mean component scores of components (PCA) across clusters (CA). ANOVA was used to compare the mean PCA factor scores among the clusters.

A stratified analysis by sex was conducted. The interaction between sex and the quintiles of the dietary patterns in relation to CRC risk was assessed by introducing cross-product terms and incorporating them into the multivariable regression. Subgroup analysis by cancer site was also conducted. To examine the heterogeneity between rectal and colon cancer, a case-only analysis was used. The distinct subtypes were used as the dependent variables, and factor scores for each dietary pattern were included as independent variables in a logistic regression model.

## 3. Results

### 3.1. General Characteristics

Table 2 lists the sociodemographic and lifestyle factors of CRC cases and controls. Compared to the control group, a higher percentage of cases were married, regular smokers and drinkers, and lived in rural areas. Cases had a lower BMI, lower educational levels, higher income, fewer household and leisure activities, and more heavy occupation activities than the controls. In the female subgroup, cases tended to experience a later age at menarche. In addition, cases had a higher proportion of a history of cancer in first-degree relatives.

### 3.2. Principal Component Analysis

PCA identified four distinct dietary patterns in the control subjects (Table 3). The first pattern, labeled milk-egg-nut-soy dietary pattern, was characterized by high factor loadings for a variety of dairy products, eggs, nuts, legumes, soy, and soy products. The second pattern, the labeled vegetable-fruit dietary pattern, was characterized by high loadings for vegetables and fruits. The third pattern, labeled poultry-fish dietary pattern, was characterized by high loadings for white meat and a low loading for refined grains. The fourth pattern, labeled the red meat-preserved food dietary pattern, was characterized by high loadings for red meat, preserved meat, and preserved vegetables. These patterns accounted for 12.33%, 11.33%, 10.35%, and 9.14% of the variance in food intake, respectively.

After adjusting for various confounding variables, the milk-egg-nut-soy dietary pattern, the vegetable-fruit dietary pattern, and the poultry-fish dietary pattern demonstrated inverse associations with the risk of CRC. The adjusted ORs (95% CIs) were 0.51 (0.42, 0.62), 0.61 (0.51, 0.74), and 0.81 (0.68, 0.97), respectively. Conversely, the red meat-preserved food dietary pattern was found to be related to a higher risk of CRC (adjusted OR Q5 vs. Q1 2.99; 95% CI, 2.43, 3.67; *P*_trend_ < 0.001) (Table 4).

#### Stratified and Subgroup Analyses

**Figure 1:**
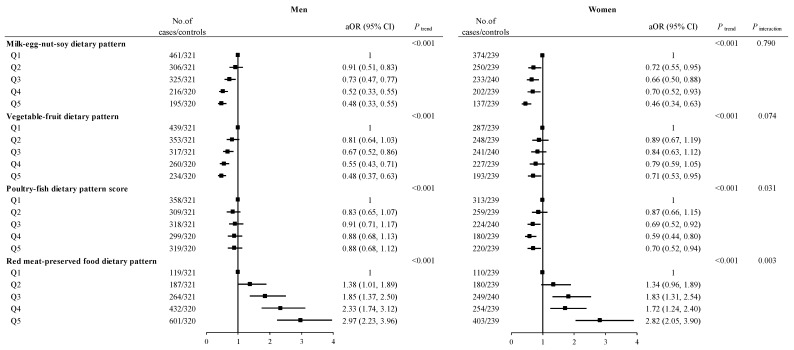
ORs and 95% CIs for colorectal cancer are stratified by sex across quintiles of factor scores. Abbreviations: Q, quintile; Q1–Q5 means the lowest quintile to the highest quintile. cOR, crude odds ratio; aOR, adjusted odds ratio; CI, confidence interval. Adjusted for age (years), sex, marital status, residence, education, occupation, income, occupational activity, MET, BMI, smoking and drinking status, history of cancer in first-degree relatives, menopausal status (woman), and daily intake of energy. The value of *P* for trend was calculated by placing Q1–Q5 as a continuous variable in the regression models.

Sex-stratified analyses showed that the milk-egg-nut-soy dietary pattern, vegetable-fruit dietary pattern, and poultry-fish dietary pattern continued to exhibit an association with a decreased risk of CRC in both men and women. However, the red meat-preserved food dietary pattern was significantly related to an elevated CRC risk in both men and women. The interactions between the poultry-fish dietary pattern (*P*_interaction_ = 0.031) and red meat-preserved food dietary pattern (*P*_interaction_ = 0.003) and sex in the risk of CRC were statistically significant (Figure 1).

**Figure 2:**
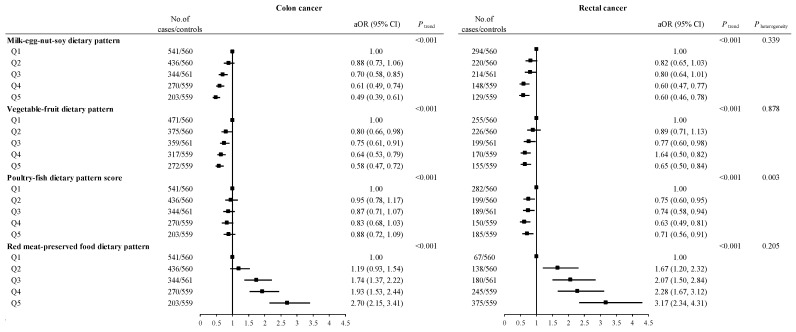
ORs and 95% CIs for colon and rectal cancer across quintiles of factor scores. Abbreviations: Q, quintile; Q1–Q5 means the lowest quintile to the highest quintile. cOR, crude odds ratio; aOR, adjusted odds ratio; CI, confidence interval. Adjusted for age (years), sex, marital status, residence, education, occupation, income, occupational activity, MET, BMI, smoking and drinking status, history of cancer in first-degree relatives, menopausal status (woman), and daily intake of energy. The value of *P* for trend was calculated by placing Q1–Q5 as a continuous variable in the regression models.

Among the cases, 1794 were diagnosed with colon cancer, and 1005 were diagnosed with rectal cancer. Subgroup analyses by cancer site revealed that the milk-egg-nut-soy dietary pattern, vegetable-fruit dietary pattern, and poultry-fish dietary pattern were all associated with a decreased CRC risk in both colon and rectal cancer (*P*_heterogeneity_ = 0.339, *P*_heterogeneity_ = 0.878, *P*_heterogeneity_ = 0.003, respectively). Conversely, the red meat-preserved food dietary pattern was associated with an increased CRC risk in both colon and rectal cancer (*P*_heterogeneity_ = 0.205) (Figure 2).

### 3.3. Cluster Analysis

Two major dietary patterns were identified using the K-means cluster analysis in the study population. It is important to note that these cluster compositions displayed a high degree of consistency in a split-half reliability analysis. Group names were assigned based on the foods and food groups with high consumption. Mean intakes (g/day) of various foods and food groups within these two dietary patterns are presented in Table 5. Most food groups exhibited significant differences between the two clusters, with the exception of salted/preserved vegetables and red meat and processed meat (*p* = 0.700 and 0.108, respectively). The first cluster, denoted as the Balanced dietary pattern, demonstrated higher mean intakes of soy products, fruits, vegetables, eggs, poultry, fish and other seafood, dairy products, nuts, legumes, and soy products. In contrast, the second cluster, labeled the Refined grain dietary pattern, was characterized by significantly higher mean intakes of refined grains.

Compared to the Refined grain dietary pattern, the Balanced dietary pattern was associated with a decreased risk of CRC (aOR, 0.59; 95%CI, 0.52, 0.66; *p* < 0.001). Even in sex-stratified analysis, the refined grain dietary pattern was consistently related to a higher CRC risk in both men and women, with a somewhat stronger association observed in men (*P*_interaction_ < 0.161). Further subgroup analysis by cancer site showed that refined grain dietary patterns had a higher risk for both colon and rectal cancer (*P*_heterogeneity_ = 0.861) (Table 6).

### 3.4. Comparison of Principal Component Analysis and Cluster Analysis

**Figure 3:**
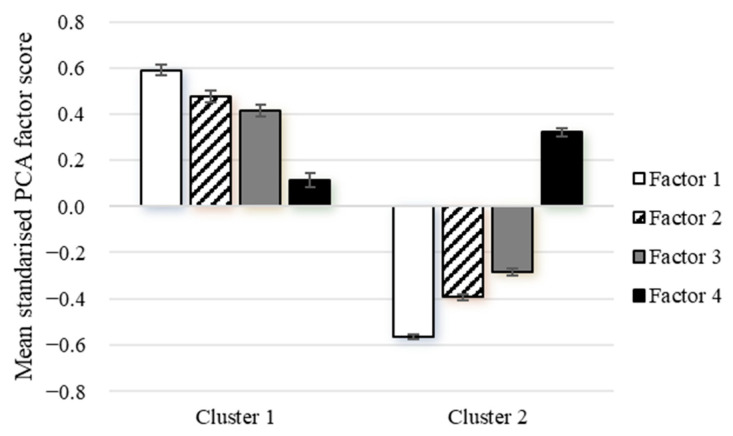
Mean (95%CI) standardized PCA factor scores for each cluster. Both factor scores and clusters were obtained from the same food frequency questionnaire variables in the same data. Cluster 1: fruits, vegetables, eggs, poultry, fish and other seafood, dairy products, nuts, and soy products; Cluster 2: refined grains; Factor 1: dairy products, eggs, nuts, soy, and soy products; Factor 2: vegetables and fruits; Factor 3: poultry, fish, and other seafood; Factor 4: red meat, processed meat, and preserved vegetables.

Figure 3 illustrates a comparison of the mean component scores of the components (PCA) across the clusters (CA). To facilitate direct comparisons, these components have been standardized to a common scale. Cluster 1 (Balanced dietary pattern) exhibited the highest score for factor 1 (mean: 0.59; 95%CI: 0.55, 0.64) and the lowest for factor 4 (0.12; 0.06, 0.18). In contrast, Cluster 2 (Refined grain dietary pattern) had the highest score for factor 4 (0.32; 0.29, 0.36) and the lowest for factor 1 (−0.57; −0.59, −0.55). These findings indicate that both CA and PCA derived remarkably similar patterns.

## 4. Discussion

The primary aim of this large-scale case–control study was to identify dietary patterns through PCA and CA and evaluate their association with CRC risk in the Chinese population. Additionally, this study aimed to explore the differences between the dietary patterns derived from these two methods. The PCA results showed a negative association between the milk-egg-nut-soy dietary pattern, the vegetable-fruit dietary pattern, and the poultry-fish dietary pattern and CRC risk. In contrast, the dietary pattern characterized by red meat and preserved foods showed a positive association with the risk of CRC. On the other hand, CA results demonstrated that, in comparison to the Refined grain dietary pattern, the Balanced dietary pattern was associated with a decreased risk of CRC. Rather than suggesting the superiority of one method over the other, our findings highlight the potential for variations in outcomes based on the chosen method for elucidating dietary patterns. Nonetheless, some fundamental characteristics of healthy diets were consistently observed across both methods. These significant associations were also observed within different sex groups and CRC subtypes.

In our study, the vegetable-fruit dietary pattern exhibited similarities with dietary patterns labeled as healthy patterns [10,12,23], vegetable and fruit patterns [11,22,28], and prudent patterns [9,18,20,27] identified in previous studies. However, our vegetable-fruit dietary pattern differed in that it did not include whole grains, a common component in the aforementioned dietary patterns. This variation may be attributed to the dietary preferences of the local population. The inverse association between the vegetable-fruit dietary pattern and CRC risk that we observed in our study aligns with findings from several studies conducted in both Western [11,20,28] and Asian countries [15,27]. However, it is important to note that not all studies have consistently yielded this result [22]. For instance, a study from the United States. Ref. [28] found that the fruit-vegetable dietary pattern, characterized by high loadings for various fruits, vegetables, and legumes such as collards, green beans, yams, and cereals, was associated with a reduced risk of CRC. Conversely, no significant association was found between the prudent pattern, which is characterized by a high intake of vegetables, legumes, fruit, whole grains, fish, and poultry, and CRC [18]. In Asian populations, a study from Korea found that the prudent pattern, which included high loadings of fruits, milk and dairy products, cereals, nuts, and a low intake of refined grains and kimchi, was inversely related to the risk of CRC [15]. And a study from Japan [27] also found that the prudent pattern, which is characterized by a high intake of vegetables, fruits, seafood, and soy foods, was associated with a decreased risk of CRC. However, another Japanese study [10] did not observe a significant association between the healthy dietary pattern, which is heavily loaded with vegetables, fruits, soy products, seaweeds, mushrooms, milk, beans, and yogurt, and CRC risk. Similarly, a study from Singapore [24] did not identify an association between the vegetable-fruit-soy pattern, characterized by vegetable, fruit, and soy food intake, and CRC risk. The derived vegetable-fruit dietary pattern mirrors the common dietary characteristics in southern China, featuring a high intake of vegetables and fruits [42]. Both of these foods are rich in potential anticarcinogenic compounds, such as fiber, folate, other B vitamins, minerals, and antioxidants. There is substantial evidence supporting the potential chemopreventive effects of fruits and vegetables [43].

The red meat-preserved food dietary pattern identified in our study was also similar to Western dietary patterns observed in previous studies. The Western dietary pattern, characterized by a high intake of red and processed meats, sugary drinks, refined grains, desserts, and potatoes, exhibits qualitative resemblances to the dietary preferences of Western populations. In alignment with our findings, the Western dietary pattern has been reported to be positively associated with CRC risk in the majority of cohorts from the United States and Western European regions [9,22,23], although exceptions exist [18]. However, in a Japanese cohort, no association was observed between the Western pattern, which was heavily loaded with meat, poultry, cheese, bread, and butter, and CRC risk [10]. A meta-analysis examining meat consumption and CRC risk suggested that higher intakes of red meat and processed meat are associated with an increased risk of CRC [44]. Studies have indicated that the consumption of processed or red meat, especially when cooked at high temperatures, may be associated with an increased risk of CRC [45]. However, a prospective cohort study showed that there was no statistically significant association between red or processed meat intake and the risk of CRC [46]. It is worth noting that the inclusion of salted/preserved vegetables as a unique component in our red meat-preserved food dietary pattern could also contribute to the increased risk of CRC. In contrast to Western countries, highly salted foods are commonly consumed in some Southeast Asian countries, such as Japan [47], Korea [15], and China [33]. A study carried out in Qingdao, China, demonstrated an association between a high-salt and pickled food pattern and an increased risk of CRC, which is in line with our results [33]. Further research is necessary to confirm the adverse effects of salted/preserved vegetables on CRC risk.

Despite the considerable diversity in specific food types across various regions, the use of different FFQs, and researchers’ varying choices regarding food groupings and component quantity retention, the dietary pattern characterized by high fruit and vegetable intake, as well as those marked by elevated red and processed meat and refined grain intake, was consistently prevalent. Given the widespread geographic and temporal consistency in the PCA results, it is reasonable to draw the conclusion that the observed patterns involving fruit and vegetables, red meat, and refined grain are not likely the result of chance observations. Instead, they appear to represent genuine, underlying dietary patterns that have been observed in many populations over time. These patterns effectively capture significant dimensions of the dietary practices of the population in Guangdong, China.

Our study revealed that the milk-egg-nut-soy dietary pattern was associated with a reduced risk of CRC. Understanding the relationship between the individual components of the milk-egg-nut-soy dietary pattern and CRC risk could provide insights into our findings. A meta-analysis [48] identified a significant association between high intake of milk and dairy products and a reduced risk of CRC compared to low intake. Another meta-analysis [49], which summarized evidence on the association between the intake of 12 major food groups and CRC risk, observed a decreased risk of CRC with a high intake of dairy. Concerning nut intake, a decreased risk was observed for colon cancer only. Soy and soy products did not exhibit statistically significant associations with CRC risk, and the relationship between egg consumption and CRC risk was not clear. Analyzing individual foods or food groups alone does not fully account for the complexity and potential interactions among various dietary components in the context of the broader spectrum of diet-disease associations.

In our study, we found an inverse relationship between the poultry-fish dietary pattern, characterized by a high intake of poultry, fish, and other seafood, and CRC risk. Fish and poultry are often components of healthy or prudent dietary patterns, which have been associated with a reduced risk of CRC in previous studies [14,16,20]. Our findings may be attributed to the independent effects of fish, poultry, or dietary interactions. Previous studies have also suggested favorable associations with poultry and fish intake [50,51,52], with some exceptions [53]. Results from research involving three large cohorts in the United States indicated that substituting white meat for red meat significantly decreased the risk of CRC [54]. Additionally, a previous study we conducted within a population in Guangdong, China, reported that a higher intake of fresh fish, including both freshwater and sea fish, was associated with a lower risk of CRC [55]. A meta-analysis [56] similarly highlighted a significant inverse association between poultry intake and CRC risk. It is worth noting that fish and poultry play a significant role in the traditional diet of coastal regions in mainland China. Residents of Guangdong province tend to consume high quantities of fish and poultry, and their food preferences lean towards lower salt and oil content, with a preference for steaming and boiling as cooking methods [57]. These dietary habits may contribute to the reduced risk of CRC.

CA is a method that categorizes individuals into relatively homogeneous groups, allowing for direct comparisons between distinct groups. Our study found that cluster 1 (Balanced dietary pattern) was associated with a decreased risk of CRC compared to cluster 2 (Refined grain dietary pattern). Our findings align with previous studies [29,30,31]. For example, a recent case–control study conducted in Morocco [29] identified two clusters, labeled ‘prudent’ and ‘dangerous’, representing the two primary dietary patterns associated with CRC. They found that individuals belonging to the "dangerous pattern" have a higher risk of developing CRC, with an OR (95% CI) of 1.59 (1.37, 1.38). In a prospective cohort study conducted in the United States [30], which categorized participants into four male clusters and three female clusters, a significant 15% lower CRC risk was observed in men when comparing the ‘fruit and vegetables’ cluster to the ‘many foods’ cluster, while a nonsignificant 10% lower risk was noted in women. A French case-control study [31] included a ‘cluster 1′ characterized by a low-energy diet. In comparison, ‘cluster 2′, defined as having a high-starch, high-fat, and low-fruit diet, was significantly associated with a higher risk of CRC, while other clusters did not exhibit an association with CRC risk. It is important to note that the definition of low-risk and high-risk dietary patterns varies among these previous observational studies. Nevertheless, the similarities are that clusters with a high intake of red and processed meat, refined grains, and sugar-rich foods are linked to a higher risk of CRC, while those with a high intake of vegetables, fruits, white meats, and dairy products are associated with a lower risk of CRC.

When comparing the dietary patterns derived from PCA and CA, we found that the food groups in the milk-egg-nut-soy dietary pattern, vegetable-fruit dietary pattern, and poultry-fish dietary pattern derived from PCA largely overlapped with the food groups in the Balanced dietary pattern obtained through CA. Notably, all these dietary patterns were associated with a decreased risk of CRC. These findings indicate that PCA and CA reveal similar underlying patterns with comparable associations regarding CRC risk. Conversely, the red meat-preserved food dietary pattern identified by PCA and the Refined grain dietary pattern identified by CA allowed us to delve deeper into dietary factors potentially associated with an increased CRC risk. PCA and CA together provide insights into dietary patterns linked to a reduced risk of CRC—those characterized by high fruits and vegetable consumption; high-quality protein sources; and lower fat content—as well as patterns tied to an increased risk; including diets rich in red meat; preserved foods; and refined grains.

Stratified analyses by sex revealed that the association between dietary patterns and CRC risk in both men and women was consistent with the findings from the overall population. Notably, the red meat-preserved food dietary pattern identified through PCA and the Refined grain dietary pattern identified through CA indicated a higher probability of increased CRC risk in men compared to women. A study from Japan showed that a prudent dietary pattern was associated with a decreased risk of CRC in men; however, the association was not statistically significant in women [19]. In a study from the United States, dietary patterns derived through CA showed that a vegetable and fruit pattern was associated with a reduced CRC risk in men, but the association did not reach statistical significance in women [30]. This variation in our study might be explained by women’s tendency to prioritize healthier food choices in their daily lives and the diverse characteristics of this study samples [58]. Additionally, this difference may stem from inherent distinctions in how men and women complete the FFQ or perhaps variations in the etiology of CRC between the two sexes [19].

In our study, the association between each dietary pattern and CRC risk remained consistent across different cancer sites. Notably, a North Carolina Colon Cancer Study conducted among Caucasian participants found that a dietary pattern rich in fruits and coarse grains was associated with a reduced risk of rectal cancer [21]. Similarly, in a prospective study involving the Japanese population, a positive association between the traditional dietary pattern and the risk of colon cancer was more pronounced among women with proximal colon cancer, while the positive association between the Western dietary pattern and the risk of colon cancer was more evident among women with distal colon cancer [10]. An American study found that the association between a Western diet and CRC appeared to be more pronounced for distal colon and rectal tumors compared to proximal colon tumors, although the formal statistical test for heterogeneity was not significant [16]. Further research is needed to clarify this issue.

Our research possesses some notable strengths. Firstly, this is the first study in China to examine the association between dietary patterns and CRC risk using both CA and PCA methods. This combined approach enhances our ability to scrutinize this association. Secondly, this study had a large sample size, ensuring robust statistical power to detect meaningful associations and conduct stratified analyses. Thirdly, the participation rate was high, which might reduce selection biases and improve the overall credibility of the research. Fourthly, we conducted comprehensive measurements of potentially significant confounding factors and incorporated them into our analyses. This meticulous adjustment minimizes the impact of confounding variables, thus enhancing the reliability of our findings.

Our study has certain limitations that warrant consideration. Firstly, the recruitment of CRC patients exclusively from one hospital could introduce selection bias. However, it is worth noting that Sun Yat-sen University Cancer Center is the largest cancer center in the South China region. Previous studies have demonstrated that the clinical characteristics of CRC patients from this cancer center were comparable to those from other major hospitals in Guangdong Province [59] and across the country [60]. Furthermore, to some extent, the high participation rate (94.91% for cases and 86.25% for controls, respectively) also helped to reduce selection bias. Secondly, information bias was a potential issue in our study. Nondifferential misclassification might attenuate the actual association between dietary patterns and CRC risk. To mitigate recall bias, we specifically selected incident CRC cases who were interviewed within three months of diagnosis. Additionally, we used food photographs depicting typical portion sizes to assist participants in accurately estimating their food consumption. Thirdly, certain decisions made during the PCA and CA processes are inherently subjective, including the selection of the final pattern solution during the extraction process [61]. In our study, efforts were made to minimize such subjectivity. For example, the FFQ foods were grouped based on approaches used in previous literature. Established criteria and best practices were applied to determine the dietary patterns. In cluster analysis, to verify the reliability of the clustering results, we conducted a 50% random split of the sample and repeated the clustering process for each of the two subsets. Fourthly, dietary patterns can exhibit local or national variations due to diverse dietary cultures and customs among different populations. When considering food composition, Western and Asian populations may display slight variations in their dietary patterns, potentially making it challenging to generalize findings to other populations. Fifthly, our study design inherently presents certain unavoidable limitations. Exploring the health effects of long-term exposure within a case-control study, where participants may have varying exposure durations (adhering to a certain dietary pattern for different lengths of time), is challenging and susceptible to bias. Furthermore, the retrospective nature of case-control studies complicates the determination of the timing of exposures and outcomes, therefore precluding the demonstration of a causal relationship between dietary patterns and CRC risk. Finally, despite correcting for these factors, there may be additional unaccounted confounders that could potentially affect the results.

## 5. Conclusions

In summary, irrespective of the method used to derive dietary patterns, our findings consistently indicate that dietary patterns characterized by higher consumption of fruits, vegetables, eggs, dairy products, nuts, soy products, and white meat are significantly associated with a reduced risk of CRC. In contrast, dietary patterns dominated by red meat, processed foods, and refined grains exhibit a positive association with CRC risk. These results contribute valuable insights to the limited body of literature on dietary patterns and their association with CRC in the Chinese population. Identifying modifiable risk factors, particularly dietary factors that have been postulated to play an important role in colorectal carcinogenesis, remains critical for the development of primary prevention strategies. Moreover, the analysis of dietary patterns holds substantial promise for enhancing dietary recommendations and public health efforts, ultimately fostering more effective dietary interventions. Our findings provide a solid theoretical foundation for CRC prevention through dietary means in the Chinese population.

## Figures and Tables

**Table 1:** Food groups were used in the dietary pattern analysis.

Food Group	Food Items
1 Salted/preserved vegetables	Salt, mustard greens, and preserved Szechuan pickles
2 Refined grains	White rice, porridge, noodles, bread, cake, biscuits
3 Fruits	Citrus fruits, apple, pear, peach, plum, banana, grape, litchi, longan, watermelon, papaya, cantaloupe, kiwi fruit, strawberry, pineapple, mango, and durian
4 Leafy vegetables	Choy sum, kale, broccoli, choy bok choy, lettuce, spinach, watercress, macaroni, tong hao, bean sprouts, wolfberry leaves, leeks, asparagus, vine greens, bok choy, cabbage, cauliflower, and parsley
5 Cucurbitaceae and Solanaceae vegetables	Eggplant, winter melon, cucumber, zucchini, bitter gourd, squash, tomato, green and red pepper, bell pepper, pumpkin, bean curd, and bean sprouts
6 Other vegetables	White radish, green radish, carrot mushrooms, fungus, cloud ears, garlic, scallions, onions, starchy tubers, and fresh corn
7 Red meat and processed meat	Pork, beef, lamb, liver, kidney, brain, Sausage, ham, and bacon
8 Poultry	Chicken with or without skin, duck, and goose
9 Fish and other seafood	Fresh water fish, salt water fish, canned fish, preserved fish, shrimp, crab, squid, cuttle, scallops, mussels, and whelk
10 Eggs	Egg and duck egg
11 Dairy products	Whole milk, whole milk powder, skim/low-fat milk, skim/low-fat milk powder, and yoghurt
12 Nuts and legumes	Peanuts, cashews, walnuts, pistachios, sesame seeds, fresh soybeans, mung beans, and red beans
13 Soy products	Hard tofu, soft tofu, fried tofu pop, tofu curd, vegetarian chicken, bean curd pudding, and soy milk

**Table 2:** The demographic factors of colorectal cancer cases and controls.

Characteristics	Case (*n* = 2799)	Control (*n* = 2799)	*p* ^a^
Age (years), mean ± SD	57.10 ±10.27	57.05 ± 9.89	0.858
Energy(kcal/d), median (P_25_, P_75_)	1481.09 (1197.73, 1815.78)	1543.13 (1259.02, 1943.86)	<0.001
Men, *n* (%)	1603 (57.27)	1603 (57.27)	1.000
Maried, *n* (%)	2661 (95.07)	2554 (91.25)	<0.001
Rural, *n* (%)	997 (35.62)	630 (22.51)	<0.001
Educational level, *n* (%)			<0.001
Primary school or below	871 (31.12)	619 (22.12)	
Middle school	784 (28.01)	713 (25.47)	
High school/technical school	681 (24.33)	751 (26.83)	
College or above	463 (16.54)	716 (25.58)	
Occupation, *n* (%)			0.013
Administrator	395 (14.11)	475 (16.97)	
Blue collar worker	624 (22.29)	608 (21.72)	
Farmer/other	1780 (63.59)	1716 (61.31)	
Income, *n* (%)			<0.001
<2000	379 (13.54)	358 (12.79)	
2001–5000	935 (33.40)	1085 (38.76)	
5001–8000	830 (29.65)	840 (30.01)	
≥8001	655 (23.41)	516 (18.44)	
Occupational activity, *n* (%)			<0.001
Non-working	334 (11.93)	968 (34.58)	
Sedentary	798 (28.51)	587(20.97)	
Light occupational	773 (27.62)	653 (23.33)	
Moderate occupational	417 (14.90)	263 (9.40)	
Heavy activity	477 (17.04)	328 (11.72)	
MET(h/week), median (P_25_, P_75_)	27.75 (8.50, 52.50)	34.50 (16.00, 56.13)	<0.001
Ever smoker, *n* (%)	1103 (39.41)	859 (30.69)	<0.001
Passive smoker, *n* (%)	793 (28.33)	802 (28.65)	0.790
Regular drinker, *n* (%)	504 (18.01)	400 (14.29)	<0.001
BMI (kg/m^2^), mean ± SD	23.34 ± 3.28	23.56 ± 3.13	0.008
Menopausal status, ^a^ *n* (%)			0.229
Premenopausal	331 (27.68)	305 (25.50)	
Postmenopausal	865 (72.32)	891 (74.50)	
First-degree relative with cancer, *n* (%)	416 (14.86)	238 (8.50)	<0.001
Age at menarche, ^a^ mean ± SD	14.95 ± 2.12	14.60 ± 3.07	0.713

Abbreviations: BMI, body mass index; MET, metabolic equivalent task; Continuous variables were assessed by the *t* test or Wilcoxon rank-sum test, and the chi-square test was used for the comparison of categorical variables. ^a^ Only for women.

**Table 3:** Factor loadings of four dietary patterns were determined in 2799 control subjects.

Food Groups	Factor 1	Factor 2	Factor 3	Factor 4
	Milk-Egg-Nut-Soy Dietary Pattern	Vegetable-Fruit Dietary Pattern	Poultry-Fish Dietary Pattern	Red Meat-Preserved Food Dietary Pattern
Total dairy products	0.558 *	−0.052	0.031	−0.471
Eggs	0.555 *	0.015	0.008	0.002
Nuts and legumes	0.546 *	0.165	−0.004	−0.054
Soy products	0.444 *	0.037	−0.007	0.145
Cucurbitaceae and Solanaceae vegetables	0.11	0.685 *	−0.066	0.009
Other vegetables	0.299	0.642 *	0.067	−0.015
leafy vegetables	−0.311	0.574 *	0.206	0.008
Fruits	0.242	0.359 *	0.346	−0.217
Poultry	−0.052	−0.096	0.712 *	−0.075
Fish and other seafood	−0.039	0.213	0.514 *	0.028
Refined grains	−0.462	−0.07	−0.503 *	−0.193
Salted/preserved vegetables	0.112	0.077	−0.182	0.709 *
Red meat and processed meat	0.022	−0.192	0.34	0.59 *

* The food groups indicate that they have a major contribution to the pattern.

**Table 4:** ORs and 95% CIs of colorectal cancer across quintiles of dietary pattern scores.

Dietary Patterns	Q1	Q2	Q3	Q4	Q5	*P*_trend_^b^
**Milk-egg-nut-soy dietary pattern**						
No. of cases/controls	835/560	656/560	558/561	418/559	332/559	
cOR (95% CI)	1	0.79 (0.67, 0.92)	0.67 (0.57, 0.78)	0.50 (0.43, 0.59)	0.40 (0.34, 0.47)	<0.001
aOR (95% CI) ^a^	1	0.85 (0.72, 1.01)	0.74 (0.62, 0.87)	0.60 (0.50, 0.72)	0.51 (0.42, 0.62)	<0.001
**Vegetable-fruit dietary pattern**						
No. of cases/controls	726/560	601/560	558/561	487/559	427/559	
cOR (95% CI)	1	0.83 (0.71, 0.97)	0.77 (0.65, 0.90)	0.67 (0.57, 0.79)	0.59 (0.50, 0.70)	<0.001
aOR (95% CI) ^a^	1	0.84 (0.70, 1.00)	0.75 (0.63, 0.90)	0.65 (0.54, 0.78)	0.61 (0.51, 0.74)	<0.001
**Poultry-fish dietary pattern**						
No. of cases/controls	671/560	568/560	542/561	479/559	539/559	
cOR (95% CI)	1	0.85 (0.72, 1.00)	0.81 (0.69, 0.95)	0.72 (0.61, 0.84)	0.81 (0.68, 0.95)	0.002
aOR (95% CI) ^a^	1	0.86 (0.72, 1.03)	0.82 (0.68, 0.98)	0.74 (0.62, 0.90)	0.81 (0.68, 0.97)	<0.001
**Red meat-preserved food dietary pattern**						
No. of cases/controls	229/560	367/560	513/561	686/559	1004/559	
cOR (95% CI)	1	1.60 (1.31, 1.96)	2.24 (1.84, 2.72)	3.00 (2.48, 3.63)	4.39 (3.65, 5.29)	<0.001
aOR (95% CI) ^a^	1	1.36 (1.09, 1.70)	1.88 (1.52, 2.33)	2.08 (1.69, 2.56)	2.99 (2.43, 3.67)	<0.001

Abbreviations: Q, quintile; Q1–Q5 means the lowest quintile to the highest quintile. cOR, crude odds ratio; aOR, adjusted odds ratio; CI, confidence interval. ^a^ Adjusted for age (years), sex, marital status, residence, education, occupation, income, occupational activity, MET, BMI, smoking and drinking status, history of cancer in first-degree relatives, and daily intake of energy. ^b^ The value of *p* for trend was calculated by placing Q1–Q5 as a continuous variable in the regression models.

**Table 5:** Mean and 95% CI of the daily food consumption frequency by the two clusters.

Food Groups (g/day)	Cluster 1 (Balanced Dietary Pattern)	Cluster 2 (Refined Grain Dietary Pattern)	*p*
	*n* = 2006 (36%)	*n* = 3592 (64%)	
Refined grains	261.93 (259.14, 264.73)	327.08 (324.63, 329.53)	<0.001
Soy products	42.44 (40.02, 44.86)	18.93 (18.19, 19.67)	<0.001
Leafy vegetables	326.72 (318.18, 335.26)	275.46 (270.64, 280.27)	<0.001
Cucurbitaceae and Solanaceae vegetables	124.53 (120.71, 128.36)	73.99 (72.41, 75.56)	<0.001
Other vegetables	107.51 (104.36, 110.65)	51.55 (50.44, 52.66)	<0.001
Salted/preserved vegetables	8.14 (7.16, 9.11)	8.37 (7.67, 9.08)	0.700
Fruits	183.80 (178.40, 189.19)	91.66 (89.36, 93.97)	<0.001
Red meat and processed meat	132.49 (126.60, 138.38)	137.56 (134.32, 140.81)	0.108
Poultry	37.01 (35.48, 38.54)	24.35 (23.59, 25.11)	<0.001
Fish and other seafood	94.09 (89.98, 98.20)	59.60 (57.60, 61.60)	<0.001
Eggs	33.42 (32.42, 34.42)	18.14 (17.65, 18.63)	<0.001
Total dairy products	90.06 (85.41, 94.71)	21.44 (20.01, 22.87)	<0.001
Nuts and legumes	13.94 (13.19, 14.68)	5.24 (5.01, 5.47)	<0.001

All values are mean and 95%CI.

**Table 6:** ORs and 95% CIs for colorectal cancer according to the two clusters.

	Cluster 1 (Balanced Dietary Pattern)	Cluster 2 (Refined Grain Dietary Pattern)	*p*	*P*_interaction/heterogeneity_
Total (*n* = 5598)				
No. of cases/controls	777/1229	2022/1570		
cOR (95% CI)	0.49 (0.44, 0.55)	1	<0.001	
aOR (95% CI) ^a^	0.59 (0.52, 0.66)	1	<0.001	
Men (*n* = 3206)				0.161
No. of cases/controls	409/687	1104/916		
cOR (95% CI)	0.46 (0.39, 0.53)	1	<0.001	
aOR (95% CI) ^a^	0.49 (0.41, 0.59)	1	<0.001	
Women (*n* = 2392)				
No. of cases/controls	368/542	828/654		
cOR (95% CI)	0.54 (0.45, 0.63)	1	<0.001	
aOR (95% CI) ^a^	0.67 (0.55, 0.81)	1	<0.001	
Colon cancer (*n* = 1794)				0.861
No. of cases/controls	500/1229	1294/1570		
cOR (95% CI)	0.49 (0.44, 0.56)	1	<0.001	
aOR (95% CI) ^a^	0.57 (0.50, 0.66)	1	<0.001	
Rectal cancer (*n* = 1005)				
No. of cases/controls	265/1229	740/1570		
cOR (95% CI)	0.49 (0.42, 0.57)	1	<0.001	
aOR (95% CI) ^a^	0.62 (0.52, 0.74)	1	<0.001	

Abbreviations: cOR, crude odds ratio; aOR, adjusted odds ratio; CI, confidence interval. ^a^ Adjusted for age (years), sex, marital status, residence, education, occupation, income, occupational activity, MET, BMI, smoking and drinking status, history of cancer in first-degree relatives, and daily intake of energy.

## Data Availability

The data that supports the findings of our study are available from the corresponding author upon reasonable request.
